# Distance education during the COVID-19 outbreak: A cross-sectional study among medical students in North of Jordan

**DOI:** 10.1016/j.amsu.2020.09.036

**Published:** 2020-10-02

**Authors:** Amer Mahmoud Sindiani, Nail Obeidat, Eman Alshdaifat, Lina Elsalem, Mustafa M. Alwani, Hasan Rawashdeh, Ahmad S. Fares, Tamara Alalawne, Loai Issa Tawalbeh

**Affiliations:** a Department of Obstetrics and Gynecology, Faculty of Medicine, Jordan University of Science and Technology, Irbid, Jordan; b Department of Obstetrics and Gynecology, Faculty of Medicine, Yarmouk University, Irbid, Jordan; c Department of Pharmacology, Faculty of Medicine, Jordan University of Science and Technology, Irbi, Jordan; d Medical Students, Faculty of Medicine, Jordan University of Science and Technology, Irbid, Jordan; e Princess Salma, Faculty of Nursing, Al-al-Bayt University, Jordan

**Keywords:** Covid-19, Online learning, Medical students, Jordan

## Abstract

**Introduction:**

In the spot of the new emerging COVID-19 pandemic and its major impact worldwide on day-to-day activities many rules had to be changed in order to fight this pandemic. Lockdown started in Jordan and around the globe affecting several aspects of life including economy, education, entertainment, and government policies. Regarding education, the priority was to ensure the safety and progress of the educational process. Thus, new methods of teaching had to be applied using the online learning at Jordan University of Science and Technology (JUST), Faculty of Medicine. This study was done to assess (1) Class Experience (2) Students and Lecturers' Interaction (3) Online Learning Advantages & Disadvantages (4) Students’ Preference.

**Methods:**

A cross sectional study was conducted Convenience sampling technique was used to collect the data from the participants using a survey composed of 18 questions on Google Forms platform. A link was sent to the undergraduate medical students at the Jordan University of Science & Technology via their e-learning accounts (n = 3700). The form was available from May 22nd, 2020 to May 30th, 2020 for 8 days long. Data analysis was done using SPSS V 23.

**Results:**

2212 out of 3700 students responded, (55.8%) of them were in the basic years and (44.2%) of them were in the clinical years. (55.8%) of students started to take online lectures after 3 weeks. (45.7%) used the hybrid teaching method (asynchronous and synchronous), (31.4%) used live classes, and 22.8% recorded classes. Zoom was the most used platform. (48.7%) and (57%) of clinical students and basic students express their interaction as bad, while the others had good and excellent interaction. Maintaining social distance was the most advantage of online teaching, while poor technical setup and no direct contact were the most disadvantage, furthermore inability to have real clinical access was a significant problem for clinical students (p < .001). With reference to students’ preferences 75% of students were not pleased with their experience and 42% of students prefer to integrate online learning with traditional learning.

**Conclusion:**

Most medical students at JUST preferred the traditional face-to-face teaching method over the solo online teaching methods with recommendations to convert to a more integrated educational system. Also, a well-established infrastructure should be done in involving online teaching.

## Highlights

• The traditional teaching is preferred more than the online one among the students.
• 43.9% of clinical group students believe that inability to have clinical access is a major obstacle when using the online learning.
• Students who had underwent online learning before the pandemic are more pleased with their online experience.
• Online learning helps maintaining social distance among students.

## 1 Introduction

In December 2019, an outbreak of pneumonia cases of an unknown etiology was recorded in the city of Wuhan in Hubei province, China. Later, the causative agent was identified as a SARS-CoV-2 (COVID-19) virus, leading to a worldwide outbreak of COVID-19 virus which was classified as a global pandemic from the World Health Organization WHO on March 11th, 2020 [1]. After that, most countries started to lockdown progressively to control the outbreak by isolating cases and tracing contacts [2].

Until June 20th, 2020 COVID-19 infected more than 8.5 million and killed 460 thousand people worldwide. Locally, Jordan reported 1015 cases with 722 recovered patients and 9 deaths [3].

On March 1st, 2020, the first case of COVID-19 was diagnosed in Jordan for a citizen who recently returned from Italy. The pandemic is expected to have enormous economic consequences as well as a marked impact on global education. On March 15th, 2020, The Hashemite Kingdom of Jordan prime ministry issued an order to suspend studying at universities and schools. The order also included closing mosques and churches, shutting down the borders, and suspending all incoming/outgoing flights, in preparation to start a curfew on March 23rd, 2020 under the supervision of Jordan's Military force and National Defence Law [4].

In order to remedy education issues, The Ministry of Higher Education and Scientific Research issued new recommendations toward converting to online teaching in the universities, setting up the way to a new method of learning in Jordan University of Science and Technology (JUST), the leading university in North of Jordan.

The faculty of Medicine at JUST has approximately 3700 on-seat students, the curriculum of medicine classify the students into two main levels: Basic science and Clinical practice groups depending on their year of study, the main focus of the first three years is studying basic science and organ systems while the remaining three years are concerned with clinical training through teaching the art of medicine practice, contributing to 6 years of studying in total.

All the lectures of basic sciences are given by face-to-face methods in the classrooms at JUST, while student's clinical training is provided at King Abdullah University as the main hospital, in addition to other peripheral hospitals in the north of Jordan. It is worth mentioning that King Abdullah University Hospital is the official assigned hospital from the Ministry of Health to admit and treat COVID-19 patients in the north of Jordan [5].

With the emergence of education and learning technologies, new platforms for remote studying were innovated making online study much easier, Google and Microsoft announced Google Meet, and Microsoft team since 2017 respectively, leading to a new era of mass virtual meetings [6,7].bib7

In addition, the online E-learning platform such as Moodle (our university E-learning) and Google Classroom, contributed to the sharing of study material, holding small discussions and contacting the lecturer at any time.

Most companies are using the online courses platform to improve their staff abilities by preloaded courses on several websites for example Udacity, Kajabi, and Datacamp. In addition, some universities such as Harvard University hosts online courses on their websites. It is believed that global online E-learning is growing by 19% annually [8].

Online learning helps saving up to 60% of traditional learning time, decreasing costs by millions of dollars, saving the environment, and developing more interactive ways [8].

As a part of Faculty vision and mission to deliver the most creative, innovative and high-quality teaching method to our students, providing permanent recommendations to develop the teaching and learning methods, it was a chance to experience a new method of remote teaching using the online learning for all the courses with no exception during these critical circumstances to ensure the progress and safety of teaching practice without any interruption.

To meet our objectives, we should understand the students' preferences during this period of remote teaching, in order to engage the students in developing the remote teaching process to be able to achieve the most satisfying and reliable results that balance between students’ preference and teaching quality, thus we have conducted this study.

## 2 Methods

### 2.1 Design

A cross-sectional study was conducted in order to asses (1) Class Experience (2) Students and Lecturers Interaction (3) Online Learning Advantages & Disadvantages (4) Students’ Preference among medical students at JUST during the period of COVID-19 pandemic.

### 2.2 Sample

A convenience sampling technique was used to utilize the sample of this study, in which an electronic survey was set on the Google Forms platform. A link was sent to the undergraduate medical students at the Jordan University of Science & Technology via their e-learning accounts (n = 3700). The inclusion criteria includes any on-seat undergraduate medical student who experienced online teaching during COVID-19 pandemic.

A Population proportion equation was used to figure out the recommended sample size with margin of error 1%.

### 2.3 Instrument

A survey of 18 questions was submitted to students using Google Forms online platform. Responding to all 18 multiple choice questions was a prerequisite for submission and recording the response in which two of them depends on the type of classes.

### 2.4 Data collection

The form was available from May 22nd, 2020 to May 30th, 2020 for 8 days long. A follow up reminder was sent after two and five days.

### 2.5 Ethical consideration

In order to collect participants data, ethical approval was obtained from the JUST Institutional Research Board (IRB approval number: 219/132/2020). Research Registry was done (UIN: researchregistry5912) in accordance to Helsinki declaration.

### 2.6 Data analysis

Data was exported in. CSV file from Google Forms, then processed to be used by IBM SPSS STATISTICS V23 for data analysis.

A Pearson Chi-Square analysis p-value <.05 used to exhibit the suggested statically difference among categorical parameters. A STROCSS guidelines used to report our study [21].

## 3 Results

### 3.1 Participants’ data

Overall, 2212 out of 3700 students at JUST faculty of medicine participated in this survey. The distribution of students was as follows: 554 (26.2%) in 1st year, 316 (15%) in 2 nd year, 309 (14.6%) in 3rd year, 375 (17.8%) in 4th year, 256 (12.1%) in the 5th year, and 302 (14.3%) in the 6th year. Those groups had subdivided into two groups: Basic's group including the first three years in which the basic courses are given and Clinical's group including the remaining years where the clinical rotations take place, contributing to 1179 (55.8%) and 933 (44.2%) of the respondents, respectively. Most of these students had not experienced online teaching before as indicated in Table 1.

**Table 1:** The respondent's distribution and their previous online teaching experience.

Survey Questions	Response options	n (%) Total n = 2112
Academic Year	1st Year (Basic)	554 (26.2)
	2nd Year (Basic)	316 (15)
	3rd Year (Basic)	309 (14.6)
	4th Year (Clinical)	375 (17.8)
	5th Year (Clinical)	256 (12.1)
	6th Year (Clinical)	302 (14.3)
Have you ever experienced online teaching before COVID-19 pandemic?	Yes	1554 (73.6)
	No	558 (26.4)

### 3.2 class experience

The majority of the students (55.8%) started to take online lectures after 3 weeks of lockdown, while (31.1%) started during the 2 nd week of the lockdown, and (13.1%) started from the 1st week of the lockdown.

A Pearson Chi-Square analysis showed a significant association between the academic year divided into two main groups (Basic and Clinical) and the starting week of online learning (1st, 2nd and 3rd week), χ2 (2) = 63, (p < .001).

36.2% of students have 5 or more classes per week, while the rest have less than 5 classes. Most of them (43.7%) took between 2 and 4 classes per week.

Around 45.7% of students attended a hybrid of Live Classes (Synchronous) and Recorded Classes (Asynchronous), while 31.4% took Live Classes only, and the rest 22.8% took only Recorded Classes. As Table 2 shows.

**Table 2:** The students’ class experience.

Survey Questions	Response options	n (%) Total n = 2112
On which week of the lockdown did you start your online learning?	After that	1178 (55.8)
	During the 1st week of lockdown	277 (13.1)
	During the 2 nd week of lockdown	657 (31.1)
How many Online classes do you have in a week?	1	424 (20.1)
	2	324 (15.3)
	3	300 (14.2)
	4	300 (14.2)
	5 or more	764 (36.2)
Type of online class	Live Classes (synchronous)	664 (31.4)
	Recorded Classes (asynchronous)	482 (22.8)
	Both	966 (45.7)

**Fig. 1:**
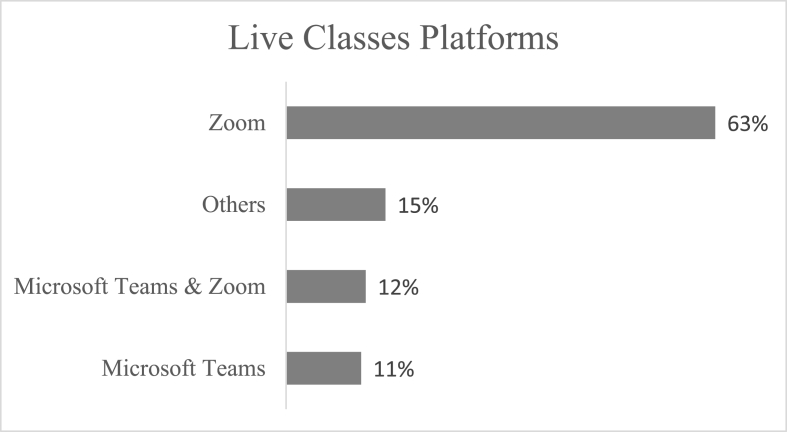
Live classes platforms.

Most of the live classes were held on well-known online meeting applications such as Zoom and Microsoft Teams. In the first place, Zoom was used in the majority of live classes (62.8%), while 11% used Microsoft Teams, 11.7% used the two aforementioned apps and 14.5% used another platform [Fig. 1].

**Fig. 2:**
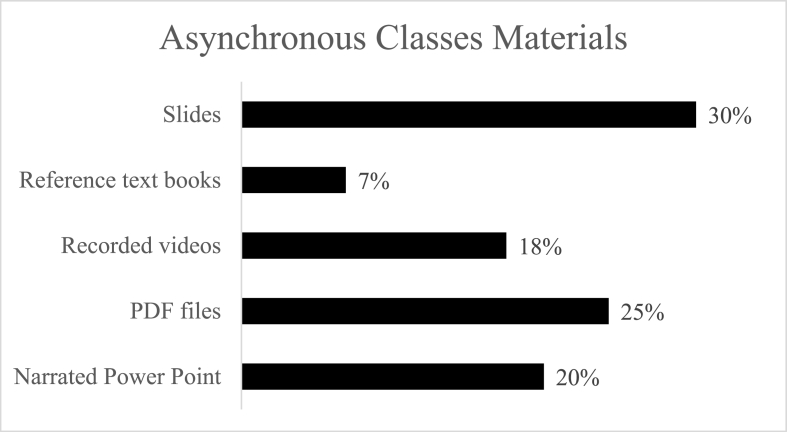
Asynchronous classes materials.

For students who attended the asynchronous classes, the most frequently used method was PDF files and Slides as a source for asynchronous classes, though the narrated PowerPoint and recorded videos were used in a less frequent manner. [Fig. 2].

### 3.3 Students and Lecturers’ Interaction

Regarding the student attendance of online classes, of all respondents, about (59%) of students were able to attend more than half of their online lectures, while the rest attended less than half of the lectures [Table 3].

**Table 3:** The students’ Attitude.

Survey Question	Response options	n (%) Total n = 2112
What is the percentage of your online classes that you were able to attend at home?	Less than 50%	850 (40.2)
	50–80%	684 (32.4)
	More than 80%	578 (27.4)

**Fig. 3:**
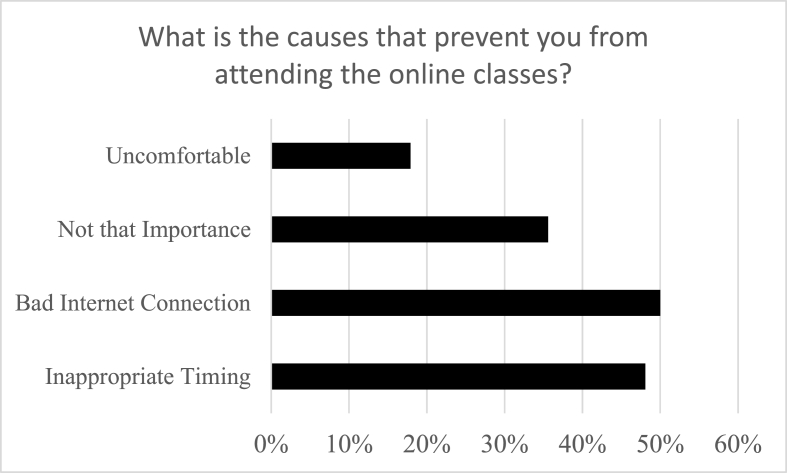
The preventive cause to attent online classes.

When asked about the causes that prevent the students from attending the online classes using multiple-choice questions, most of the students found that bad internet connection and inappropriate timing to be the main obstacle, that prevents them from attending, although about one-third of the students think that live attendance is not important, while a minority of them believe that online learning makes them uncomfortable [Fig. 3].

**Fig. 4:**
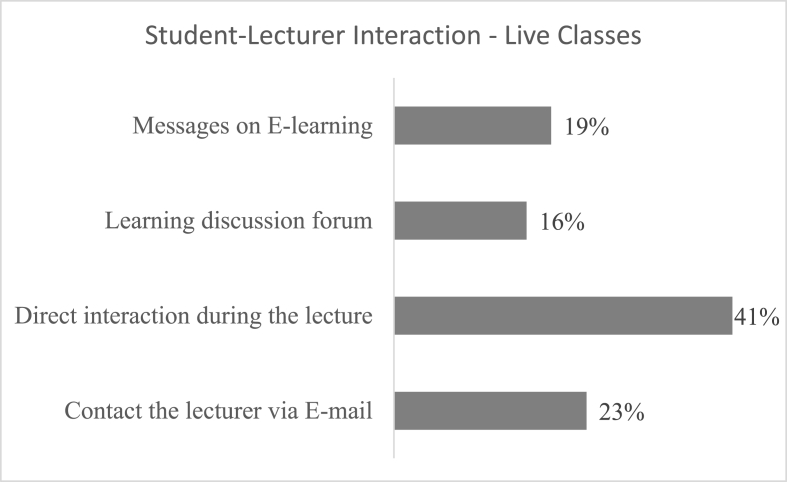
Student-lecturer interaction (live classes).

Student lecturer interaction revealed that the majority were interacting directly during live lectures, while the E-Mail and university E-learning massages were used less frequently [Fig. 4].

**Fig. 5:**
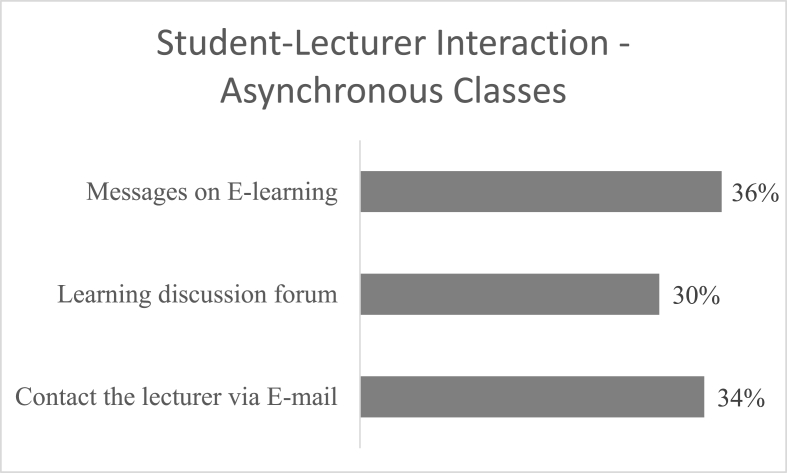
Student-lecturer interaction (asynchronous classes).

On the other hand, student-instructor interaction for asynchronous classes was through E-learning massages, University E-mail, or discussion forum on e-learning platform in an equal manner [Fig. 5].

**Fig. 6:**
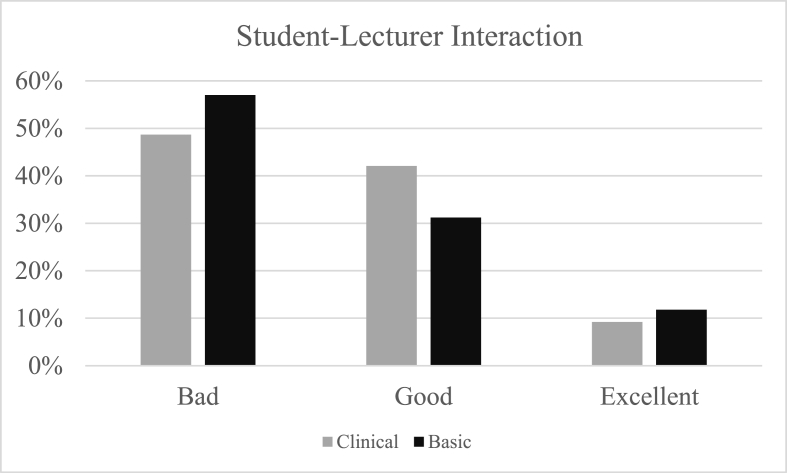
Student-lecturer interaction.

About (48.7%) of Clinical students described their interaction with the lecturer as bad, in contrast to (42.1%) and (9.2%) of them reported good and excellent interaction, respectively. However, more than half (57%) of Basic students had bad interaction, the remaining (31.2%) and (11.8%) had good and excellent interaction, respectively [Fig. 6].

A Pearson Chi-Square indicated a significant association between the academic year divided into two main groups (Basic and Clinical) and the interaction with the lecturer, χ^2^ (2) = 27.22, (p < .001).

### 3.4 Online Learning Advantages & Disadvantages

Limited social contact such as social distancing and saving money and energy from using the university transportations were the most advantages of online learning, while the second main advantage was considered as an easier method of learning. Most of the disadvantages were regarded as the need for technical setup as well as on-ground direct contact and no clinical access.

At the same time, the inability of providing a calm environment for the students during the online class and lower academic achievement was considered the second most reported disadvantage of the online learning, while the minority of disadvantages were due to inability to adapt, more absence and feeling the online class are not safe. As Table 4 manifest.

**Table 4:** Online learning advantages and disadvantages.

Survey Questions	Response options	n (%) Out of total n = 2112
Advantages of Online learning (Multi-choices)	Limited consequences of social contact	1232 (58.3)
	Saves money and energy from using transportation from and to University	1028 (48.7)
	An easier method of learning	712 (33.7)
	Less absences than traditional teaching	488 (23.1)
	Better interaction of students in classes	475 (22.5)
	Better/higher academic achievement	262 (12.4)
Disadvantages of Online learning (Multi-choices)	Needs technical means	1218 (57.7)
	No direct contact with the lecturer	964 (45.6)
	No clinical access	928 (43.9)
	Inability to provide a calm environment in the house while having the online class	768 (36.4)
	Worse/lower academic achievement	601 (28.5)
	Cannot yet adapt with Online learning	497 (23.5)
	More absences than in traditional teaching	431 (20.4)
	Feeling online classes are not safe	286 (13.5)

A Pearson Chi-Square analysis exhibits a significant difference between the academic year divided into two main groups (Basic and Clinical) and the availability of clinical access to medical students, χ^2^ (1) = 240.09, (p < .001).

### 3.5 Students’ preference

Five questions targeted the student's preference regarding their experience with the last online courses and how to improve it.

The majority of the students agreed that the lecturer and the students should have a better technical setup, whereas in the second place more improvement should be considered in class timing, class interactivity, classes privacy, and simpler ways of explanation and discussion during classes [Table 5].

**Table 5:** Online learning improvement.

Survey Question	Response options	n (%)Out of total n = 2112
What are the points that you think may improve the online learning? (Multi-choices)	For the lecturer to have a better technical setup	1171 (55.4)
	For the student to have a better technical setup	861 (40.8)
	Different classes timings	749 (35.5)
	More dynamic and interactive classes	657 (31.1)
	More private environment at student's house	638 (30.2)
	Simpler ways of explanation and discussion during classes	604 (28.6)

**Fig. 7:**
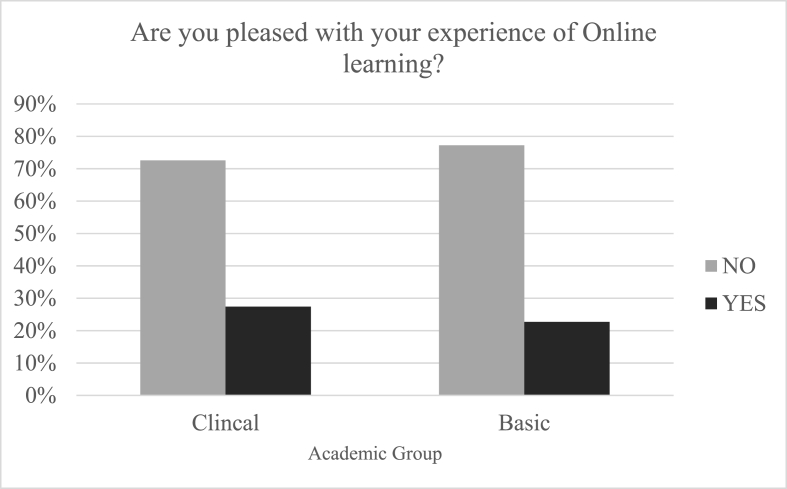
Experience satisfaction.

**Fig. 8:**
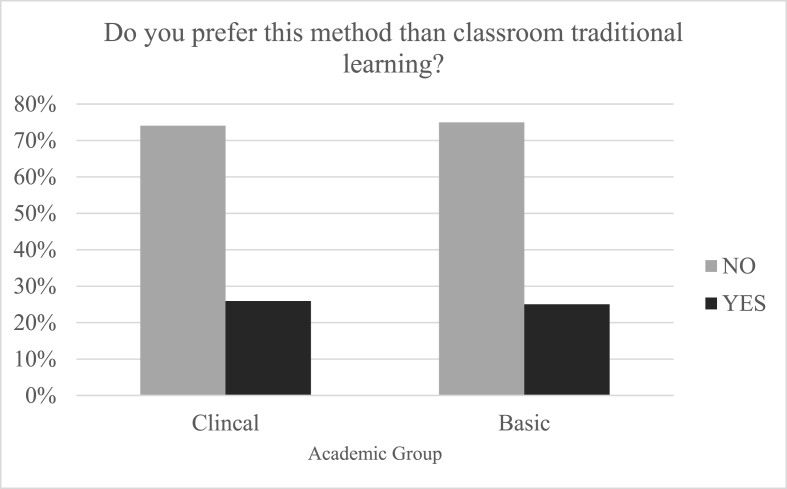
Do you prefer online learning method than classroom traditional learning?.

**Fig. 9:**
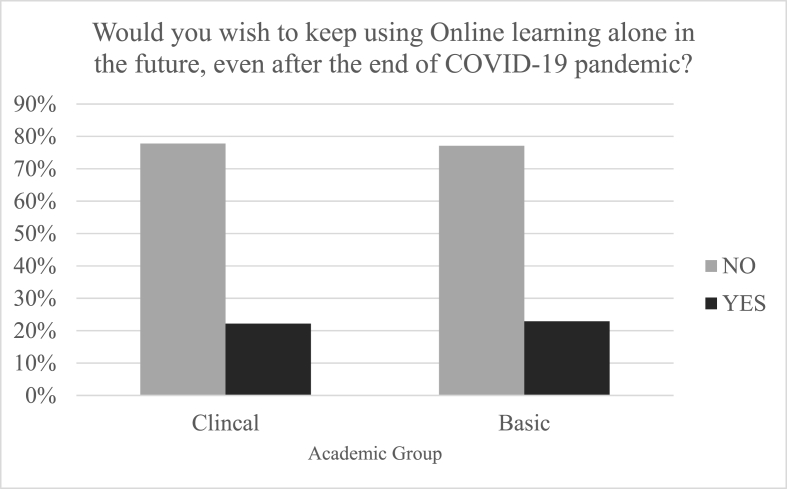
Keep using Online Learning alone.

Nearly (75%) of students (Basic and Clinical) weren't pleased with their online experience, didn't prefer this method of teaching rather than the traditional one and don't wish to use it as an official teaching method in the upcoming future [Fig. 7] [Fig. 8] [Fig. 9].

**Fig. 10:**
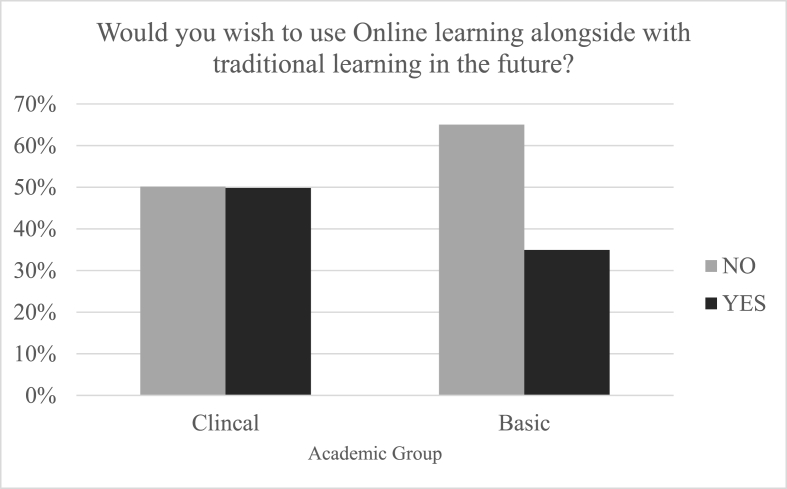
Use more Integrated System.

While about (42%) of students would prefer to integrate online learning with the traditional one after COVID-19 pandemic [Fig. 10].

A Pearson Chi-Square analysis exhibited a significant association between experienced online learning before COVID-19 and being satisfied with online learning, χ^2^ (1) = 142.731, (p < .001).

## 4 Discussion

During the last 3 years, the Faculty of Medicine at Jordan University of Sciences and Technology (JUST) committed to integrating online learning using the Moodle platform (JUST E-learning). These efforts succeeded to convert two courses MED362 MEDICAL ETHICS & MED382 HEALTH ADMINISTRATION which are part of the third-year curriculum to completely online courses using asynchronous lecture videos, online assignments and study material files, This conversion enabled the students to save their time in order to focus more on the other tough courses such as MED322 NEUROSCIENCE (1), MED332 NEUROSCIENCE (2), and MED352 URINARY & REPRODUCTIVE SYSTEM which are all part of the same semester in that year, it was an initial setup for establishing online approach in our Faculty of Medicine.

This year as COVID-19 became a pandemic and lockdown started worldwide, most of the academic facilities converted to use online learning as an alternative during this period to ensure the safety of staff and students [9]. In addition to that, some medical colleges adopted open-book examination shifting toward a new entirely system of online teaching and examination [19].

As we experienced a massive transition to online learning, it was extremely important to study the effects of online learning using several parameters on medical students especially, it is known that clinical courses need on-ground interaction for the purpose of clinical practice (physical examination, history taking and clinical skills), on the contrary, basic science courses are more flexible to be converted to online as it needs a minimal real-time interaction between the lecturer and the students.

Starting with class experience, most of the courses took more than 2 weeks to start as it took about two weeks to have a full lockdown in Jordan. Most of the students had on average 2 to 4 classes per week. Zoom was the most used platform for live classes as it was the most famous cloud meeting app during that period [10].

We noted a significant relation between clinical students and basic students due to the previous experience using the online learning before COVID-19 as clinical students had passed the Medical Ethics and Health administration online courses and have established a background about online teaching, this is consistent with Leslie Hamilton et al. of which the older students prefer to use online scientific sources such as e-books and online libraries, due to the experiential nature of their courses that requires more literature knowledge [11].

Students believe that bad internet connection and inappropriate timing was preventing them from attending the classes, it is worth to mention that Jordan telecommunication companies had suffered from a massive load on the internet network that decreased the internet speed and connection in several areas. Some of the students think that online live attendance is not important as they can study from the sources directly, this was found to be congruous with the concerns of some educators that these methods may lead to loss of fundamental knowledge such as on-hand clinical practice that provided by the educator work experience due to consuming more time focusing on things that the students should prepare themselves [12].

When asked about online learning advantages, most of the students stratify that online learning would help on maintaining social distance as they are at home and have less contact with others as well as it will save them some money [13] and energy though our university located out of Irbid city and it's far away from the capital Amman approximately 90 km. Kay D et al. believes that the online teaching provides the students with more comfortable space and increases their focus on studying since they are accessing the lectures from their homes [20].

Given that should ease and rise the expectations of student attendance. This is consistent with a study of Orthopaedic Education, though higher residents' attendance was noticed in the virtual learning sessions compared to the traditional one [18]. However, our students’ online attendance rate was below than usual. This may be due to the unestablished online teaching infrastructure and our limited experience in this field.

On the other side, most of the students stated that online learning needs more technical means. No direct contact between the lecturers and students is a significant obstacle as students and lecturers have been making a long way of on-ground interaction during the regular lectures before COVID-19, this is consistent with Friedman CP et al. [16] The lack of clinical access to medical students exhibits one of the most disadvantages for them, this owed to the essentiality of patients exposure for this period of medical training. Although, a further step should be taken to integrate more skill labs sessions in our School of Medicine as most medical schools started to change their curriculum to include more educational simulation in order to ensure the safety and quality of teaching. Ethically, this will save the patient autonomy [17].

A minimal count of students don't feel safe using online learning due to security reasons that surrounded Zoom App as it is the most used app in our online learning [10]. It is worth mentioning that JUST Faculty of Medicine will convert to use Microsoft Teams in the whole upcoming online classes for a privacy concern about the Zoom app and as the university offers free access to it by using university E-mail.

Most of the students weren't pleased with their online experience, nonetheless, a significance between students who had past online experience and being pleased with online learning stratifying some studies results suggesting that first-time online experience being substantially worse comparing to experienced students, this is congruent with Freeze R et al. [15].

We share our beliefs with other studies that the effectiveness of online learning is affected by several diverse parameters such as age, attitude, satisfaction, and level of engagement [14].

As doctors, health care is becoming more digitized in several ways such as documentations that will be recorded in electronic health records thus online learning will provide capabilities to the students to deal with the upcoming digitized medicine [16].

The use of convenience sampling technique and cross-sectional design that is based on a questionnaire are the main limitations of the current study which may affect the generalizability of the findings. Further research studies are recommended using a more representative sample of medical students from all over Jordan, applying a longitudinal design that is based on a valid and reliable tool to help improving the external validity of the results.

Finally, keeping the progress of the teaching process is of high value during this critical situation with maintaining students and lecturers’ health as our priority. COVID-19 changed the world, a high number of casualties and cases are being reported around the hour, in the other side, it helps us to discover a new way of learning by setting up the borders for a new era of online learning also it helps us to bring the world together in fighting this pandemic, we hope that this nightmare will stop as soon as possible.

## 5 Conclusion

The world is always changing and progressing whether intentional or by circumstances against our will, hence we should always aspire to move forward and try to develop our vision and tools, in the meaning time solo online education experience was not favourable amongst the majority of our students, due to various reasons, some of which we can modify, and some we can't, however, it is very important to investigate and alter our deficiencies to deliver the maximum quality of teaching. Also, we should setup a well-established infrastructure to integrate the online teaching in the correct manner based on international experiences.

## Ethical approval

IRB approval number: 219/132/2020.

## Sources of funding

No funding.

## Author contribution

**Study concept or design:** Amer Sindiani, Nail Obeidat, Mustafa Mohamed Alwani. **Data collection:** Amer Sindiani, Lina Elsalem, Eman Alshdaifat, Tamara Alalawn. **Data analysis:** Amer Sindiani, Mustafa Mohamed Alwani, Ahmad Salah Fares. **Writing the paper:** Amer Sindiani, Nail Obeidat, Mustafa Mohamed Alwani, Ahmad Salah Fares, Hasan Rawashdeh, Loai Twalbeh.

## Registration of Research Studies

1 Name of the registry: Distance education during the COVID-19 outbreak: a cross-sectional study among medical students in North of Jordan.
2 Unique Identifying number or registration ID: researchregistry5912.
3 Hyperlink to your specific registration (must be publicly accessible and will be checked): https://www.researchregistry.com/browse-the-registry#home/registrationdetails/5f36ea72735ea600152340fd/.

## Guarantor

Amer Sindiani.

## Consent

IRB has been obtained (IRB approval number: 219/132/2020).

Data has been collected using survey on Google form send to all students via e-learning and the survey did not include any personal data (name, university ID …etc).

## Declaration of competing interest

No Conflict of interest.
